# Challenges in neuropsychological improvement after shunt surgery for idiopathic normal pressure hydrocephalus

**DOI:** 10.3389/fnagi.2025.1725486

**Published:** 2025-12-12

**Authors:** Ondřej Rýdlo, Adéla Bubeníková, Petr Skalický, Klára Häcklová, Robért Leško, Aleš Vlasák, Hana Georgi, Ondřej Bradáč

**Affiliations:** 1 Department of Neurosurgery, Second Medical Faculty, Charles University and Motol University Hospital, Prague, Czechia; 2 Department of Neuroscience, First Medical Faculty, Charles University, Prague, Czechia; 3 Department of Neuropsychology, First Medical Faculty, Charles University and Military University Hospital, Prague, Czechia; 4 Prague College of Psychosocial Studies, Prague, Czechia; 5 Department of Neurosurgery and Neurooncology, First Medical Faculty, Charles University and Military University Hospital, Prague, Czechia

**Keywords:** normal pressure hydrocephalus, neuropsychology, shunt surgery, dementia, cognitive decline

## Abstract

**Objectives:**

To evaluate cognitive and mood changes 3 months after shunting for idiopathic normal-pressure hydrocephalus (iNPH), and compare postoperative outcomes with matched healthy controls across cognitive domains.

**Methods:**

Thirty-three iNPH patients underwent neuropsychological testing preoperatively and at 3 months; 71 age-, sex-, and education-matched controls were assessed once. Tests were grouped into six cognitive domains.

**Results:**

Shunting yielded significant gains in Verbal Memory and Psychomotor Pace; Executive Functions improved selectively. Non-Verbal Memory, Language, and Visuospatial Abilities showed no postoperative change. Depressive symptoms decreased significantly. However, at 3 months patients still performed worse than controls on all tests (all *p* < 0.001).

**Conclusion:**

Shunt surgery produces measurable yet domain-limited cognitive benefits in iNPH at 3 months, particularly in verbal learning and processing speed, alongside mood improvement. Performance remains below healthy norms, indicating partial recovery. Larger, prospective cohorts and longer follow-up are needed to determine durability, breadth of cognitive change, and predictors of response.

## Background

Idiopathic normal pressure hydrocephalus (iNPH) is a condition characterized typically by the Hakim’s triad: incontinence, gait disturbance and cognitive decline (Nakajima et al., 2021). It predominantly affects the elderly, with a prevalence of 1.4–3.7%, increasing with age (Andersson et al., 2019; Shprecher et al., 2008). The most prominent symptom is gait disturbance, occurring approximately in 90–100% of patients, followed by the cognitive decline occurring in 78–98% of patients (Agerskov et al., 2018; Nakajima et al., 2021). Cognitive decline in iNPH significantly impacts patients’ quality of life and functional independence. Ventriculoperitoneal shunting (VPS) is the standard treatment aimed at restoring cerebrospinal fluid (CSF) dynamics, yet the extent and mechanisms of cognitive improvement remain under investigation. In our center, diagnosis of iNPH follows a standardized multimodal protocol combining clinical and neuropsychological assessment, neuroimaging with Evans’ index > 0.3, lumbar infusion testing, and extended CSF drainage over 120 h; patients who show at least a 15% improvement on the Dutch Gait Scale together with supportive imaging criteria are classified as probable shunt responders and selected for VPS, whereas non-responders are managed with continued outpatient follow-up.

Cognitive assessment in iNPH often relies on global screening tools such as the Montreal Cognitive Assessment (MoCA) and Mini-Mental State Examination (MMSE), both of which often show postoperative improvement (Hülser et al., 2022; Xiao et al., 2022). Current guidelines recommend their use in the diagnostic work-up of iNPH as pragmatic first-line measures of global cognition (Nakajima et al., 2021) and some authors suggest cutoff scores for cognitive (non)responders (Gallina et al., 2018; Passaretti et al., 2023; Caneva et al., 2025). While these tools are useful, their predictive abilities remain limited and they are insufficient to describe a patient’s full cognitive profile or to capture more subtle, domain-specific patterns of change (Lilja-Lund et al., 2023). For this reason, comprehensive neuropsychological assessments remain essential when the aim is to characterize the cognitive phenotype of iNPH in detail and to understand which domains benefit most from cerebrospinal fluid diversion. The overall cognitive improvement may correlate with the depth and nature of cognitive deficits before surgery (Thomas et al., 2005). Regardless of the severity of the cognitive deficit, the shorter the time since the onset of symptoms, the greater the expected postoperative improvement in cognitive performance (Xiao et al., 2022). While global cognitive screening is useful, it provides limited insight into the specific neuropsychological profile of iNPH patients. Tools like the MoCA rely on abbreviated versions of comprehensive tests, often missing subtle domain-specific deficits. As a result, screenings alone cannot capture which cognitive domains are most affected or how they change post-treatment. Incorporating detailed neuropsychological assessment can therefore enhance diagnosis, treatment planning, and monitoring of cognitive changes.

The most affected cognitive domains in iNPH are executive functions (EFs), memory, and psychomotor pace (PP), with attention also showing significant impairment (Rýdlo et al., 2024; Xiao et al., 2022). On the other hand, there is inconclusiveness regarding visuo-spatial abilities (Bugalho et al., 2014; Xiao et al., 2022) and the language seems to be mostly spared (Saito et al., 2011).

EF deficits are among the most studied and appear to improve after shunting, though findings remain inconsistent (Bugalho et al., 2014; Devito et al., 2005; Hellström et al., 2008; Katzen et al., 2011; Laidet et al., 2015; Solana et al., 2012). While some studies report significant gains, others only observe statistical trends (Hülser et al., 2022). Memory dysfunction, particularly in verbal and non-verbal long-term recall, also improves postoperatively, suggesting a role for CSF restoration in cognitive recovery (Rýdlo et al., 2024). There is a significant improvement after shunt surgery in long term memory, both verbal (Hellström et al., 2008; Solana et al., 2012) and non-verbal (Skalický et al., 2022; Thomas et al., 2005). Attention, closely linked to EFs, seems more impaired in iNPH compared to Alzheimer’s disease, and some studies suggest it improves after surgery, further supporting the need for targeted cognitive rehabilitation (Hülser et al., 2022; Ogino et al., 2006; Rýdlo et al., 2024).

Despite these findings, the literature on detailed cognitive profiling in iNPH remains limited. A more precise understanding of cognitive deficits before and after shunting is crucial for refining the diagnostic process, differentiating dementia subtypes, and optimizing patient management. Therefore, this study delineates the pre- and postoperative cognitive phenotype of iNPH and identifies which functions exhibit clinically meaningful change following CSF diversion. Accordingly, we pursue two analytic aims: (i) quantify postoperative change at the domain level in iNPH and (ii) characterize the postoperative iNPH domain outcome relative to controls. This approach is intended to refine diagnostic precision, inform differential diagnosis among dementia syndromes, and optimize patient management through targeted assessment and monitoring. Future research should prioritize comprehensive neuropsychological assessments to enhance treatment strategies and improve patient outcomes.

## Materials and methods

### Participants

Between 2018 and 2021, a total of 126 patients with suspected iNPH were evaluated. They underwent a comprehensive diagnostic protocol, including clinical, psychological, imaging, and functional assessments. To be included in the study, patients had to meet the following criteria: (1) symptom onset lasting at least 3 months, (2) an Evans’ index greater than 0.3, (3) gait disturbance measured by Dutch Gait Scale along with at least one additional symptom from Hakim’s triad, and (4) no other known condition that could explain their symptoms. Admitted patients underwent lumbar infusion test (LIT) and CSF drainage for 120 h. Clinical and gait evaluations were repeated after ELD. Patients who showed ≥ 15% improvement on the Dutch Gait Scale (DGS) after ELD, in conjunction with imaging findings consistent with iNPH, were classified as shunt responders and underwent VPS implantation. Patients who did not reach this threshold were categorized as non-responders and were referred for further outpatient follow-up.

Out of the 56 participants included in the study from neurosurgical perspective, we excluded all participants with Geriatric Depression Scale (GDS-15) score 10 or higher (*n* = 3), which indicates severe depressive symptoms that can substantially interfere with cognitive performance (Shin et al., 2019). We further excluded participants with a MoCA score lower than 18 (*n* = 20). In line with previous work, this threshold was used as a pragmatic boundary for at least moderate global cognitive impairment, where floor effects and reduced test validity limit the interpretability of a detailed domain-specific neuropsychological profile (Milani et al., 2018; Yeung et al., 2020). Thus, the final neuropsychological sample represents patients with mild-to-moderate cognitive decline and without severe depressive symptoms, in whom a 2–3-h neuropsychological protocol is both feasible and clinically meaningful.

The control group consisted of 71 healthy probands who were derived from the National Normative Study of Cognitive Determinants of Healthy Aging, NANOK (Štěpánková et al., 2015). This sample was adjusted to the experimental group by age, education and gender.

### Neuropsychological battery

We used a complex neuropsychological battery that was created by trained neuropsychologists with regard to the iNPH patient cognitive profile. The battery evaluated multiple cognitive domains and the protocol of administration was fixed in order to avoid inter-patient variance or interference of for example different memory tests. For executive functions (EFs), we used the completion time of Trail Making test in form B (Bezdicek et al., 2017), the Block Design subtest of Wechsler Adult Intelligence Scale (BDT; Černochová et al., 2010), and the Letter Fluency (czech standardized version with letters N, K, P; Nikolai et al., 2015). For verbal memory (vM), we used the results of the Auditory Verbal Learning Test (AVLT; score for I-V trial, which is a measurement of memory capacity and ability to learn, VI trial and 30 min delay recall, Frydrychová et al., 2018). Similarly, we used the Rey-Osterrieth Complex Figure Test reproduction (ROCF, 3 min; Drozdová et al., 2015) and 30 min delay recall for non-verbal memory (nVM). For visuospatial abilities (VA), we used the ROCF - copy (Drozdová et al., 2015). We furthermore assessed Psychomotor Pace (PP) with Trail Making Test version A (Bezdicek et al., 2017) and language (LA) with Category fluency (Czech standardized version for animals and vegetables, Nikolai et al., 2015). Additionally, we used the Montreal Cognitive Assessment (Kopecek et al., 2017; Nasreddine et al., 2005) as a global screening of cognitive performance, which includes subtests assessing the patient’s visuoconstructional, executive functions, memory, attention, and verbal functions. Lastly, we assessed depressive symptoms with Geriatric Depression Scale (GDS-15) with 15 items, a shortened version of the original 30-items questionnaire (Yesavage et al., 1982).

### Statistical analysis

For comparing the control group with the 3 months post-shunt iNPH group, the appropriate statistical test was chosen based on the normality and homogeneity of variance assumptions. The normality of the data was analyzed with the Kolmogorov–Smirnov test. An Independent Samples t-Test was used for normally distributed data with equal variances. Within the iNPH group, paired t-tests were used to compare pre-shunt and post-shunt (3 months) scores. The magnitude of change was evaluated according to Cohen’s criteria (0.8 = large, 0.5 = medium, 0.2 = small, Sullivan and Feinn, 2012). Statistical significance will generally be considered at a threshold of *p* < 0.050. All statistical analyses were conducted with Jamovi statistical software (v2.3, The Jamovi Project 2022).^1^

## Results

**Figure 1:**
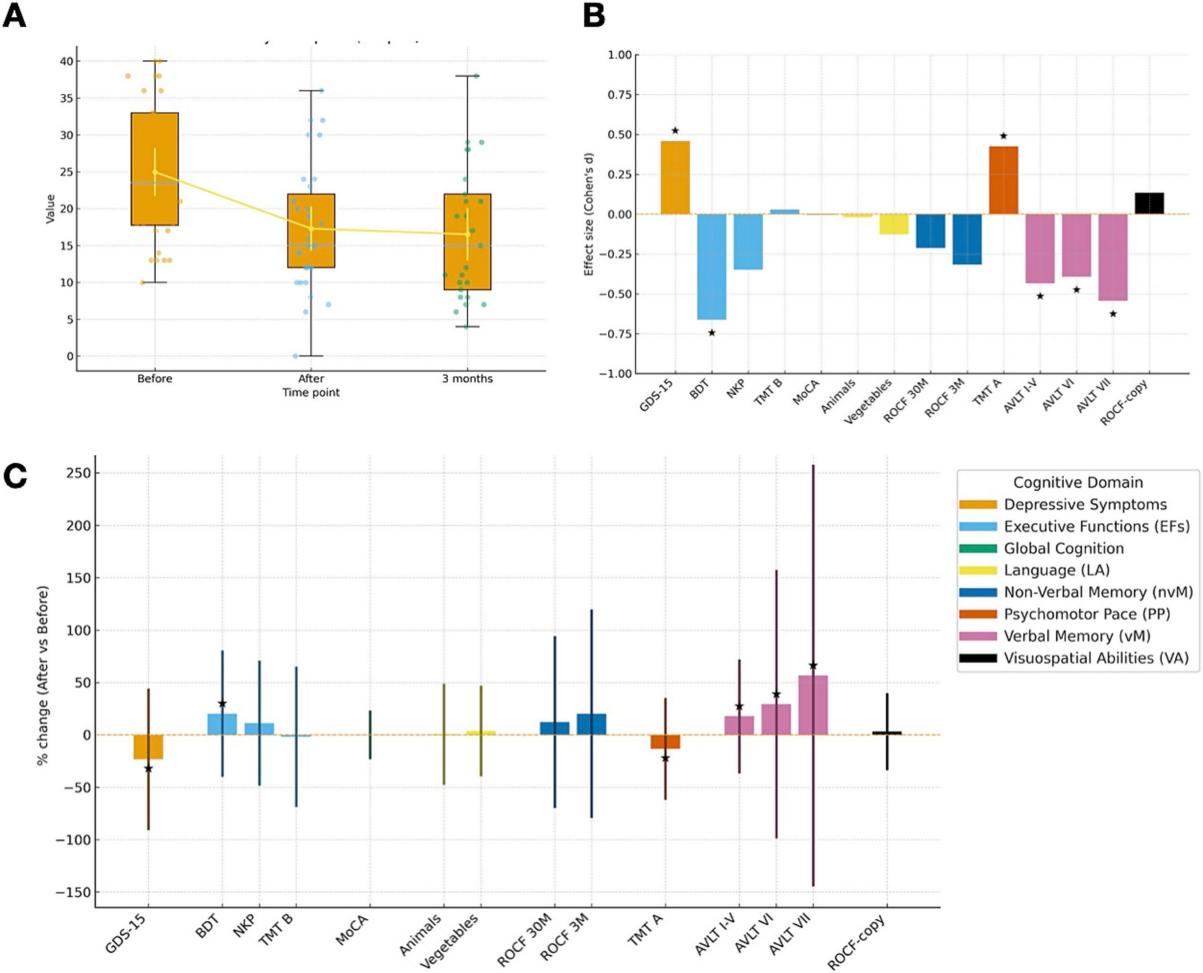
**(A)** Distribution of DGS scores across time points (Before, After, 3 months). Boxes show the median and interquartile range (IQR); whiskers extend to 1.5 × IQR. Dots are individual observations (jittered for visibility). The overlaid line with circles plots the mean ± 95% CI at each time point to highlight central trends. **(B)** Effect sizes (Cohen’s d) for pre–post change by test, color-coded by cognitive domain. Bars display Cohen’s *d* (Before–After) for each test; colors indicate domains. The horizontal zero line denotes no change; the y-axis is limited to −1.0 to +1.0 for comparability. *indicates *p* < 0.05 for the corresponding test (two-sided), based on the paired pre/post analysis reported in the text. Note that negative *d* values reflect improvement for measures where lower scores are better (e.g., TMT A/B, GDS-15), whereas positive *d* indicates improvement for higher-is-better measures. **(C)** Percent change in neuropsychological test performance (After vs. Before), grouped by cognitive domain. Bars show percent change relative to baseline for each test; colors denote cognitive domains. Error bars represent propagated SDs from pre- and post-intervention SDs via the delta method. The dashed line marks 0% change. *marks tests with *p* < 0.05. Positive values indicate improvement for higher-is-better measures (MoCA, AVLT, ROCF, NKP, BDT, Animals, Vegetables) and worsening for time/symptom measures (TMT A/B, GDS-15); interpret direction accordingly.

A total of 33 patients with the mean age of 71.3 ± 5.2 years fulfilled the diagnostic criteria and had been included in this study. Gait performance, assessed with the Dutch Gait Scale (DGS), improved significantly immediately after surgery (*p* = 0.003) and this improvement was sustained at the 3-month follow-up (*p* = 0.002; Figure 1A).

### Cognitive performance before and after shunt surgery

We analyzed cognitive performance in iNPH patients pre- and 3 months post-shunt surgery (Figure 1; Table 1). Paired-samples t-test showed no significant change in MoCA scores (*p* = 0.979). However, vM improved significantly: AVLT I-V (*p* = 0.033), AVLT VI (*p* = 0.043), and AVLT VII (*p* = 0.007). nvM did not significantly improve: ROCF 3 M (*p* = 0.100) and ROCF 30 M (*p* = 0.297).

**Table 1:** Comparison of cognitive performance of iNPH patients before and after shunt surgery.

Cognitive domain	Test	Pre-intervention (M ± SD)	Post-intervention (M ± SD)	t-value	Degree of freedom	*p*-value	Effect size (d)
Global Cognition	MoCA	21.33 ± 2.35	21.35 ± 4.36	−0.02	29	0.979	−0.004
Verbal Memory (vM)	AVLT I-V	**24.7 ± 8.68**	**29.11 ± 8.72**	**−2.26**	**26**	**0.033**	**−0.434**
	AVLT VI	**2.83 ± 2.24**	**3.66 ± 2.18**	**−2.12**	**28**	**0.043**	**−0.393**
	AVLT VII	**2.31 ± 2.36**	**3.62 ± 2.82**	**−2.93**	**28**	**0.007**	**−0.544**
Non-Verbal Memory (nvM)	ROCF 3 M	9.36 ± 6.11	11.26 ± 5.72	−1.70	28	0.100	−0.316
	ROCF 30 M	10.02 ± 5.92	11.24 ± 4.84	−1.07	24	0.297	−0.213
Executive Functions (EFs)	TMT B*	200.48 ± 74.17	197 ± 112.72	0.14	20	0.890	0.030
	NKP	25.83 ± 10.19	28.72 ± 10.48	−1.88	28	0.070	−0.349
	BDT	**20.82 ± 7.82**	**25.04 ± 8.34**	**−3.51**	**27**	**0.002**	**−0.663**
Psychomotor Pace (PP)	TMT A*	**86.55 ± 34.09**	**75.01 ± 29.96**	**2.29**	**28**	**0.030**	**0.425**
Visuospatial Abilities (VA)	ROCF-copy	27.09 ± 7.54	27.95 ± 6.26	−0.71	27	0.483	0.134
Language (LA)	Animals	14.79 ± 5.49	14.90 ± 4.50	−0.11	28	0.916	−0.020
	Vegetables	8.14 ± 2.53	8.45 ± 2.34	−0.69	28	0.498	−0.127
Depressive Symptoms	GDS-15*	**4.72 ± 2.76**	**3.62 ± 2.38**	**2.47**	**28**	**0.020**	**0.458**

MoCA, Montreal Cognitive Assessment; AVLT, Auditory Verbal Learning Test; ROCF, Rey-Osterrieth Complex Figure; TMT, Trail Making Test; GDS-15, Geriatric Depression Scale 15 items. Missing values were excluded separately analysis by analysis. Bold results indicate a statistically significant result. *Decrease in scores equals an improvement.

EFs showed mixed results. While TMT B (*p* = 0.890) and NKP (*p* = 0.070) did not reach significance, the BDT improved significantly (*p* = 0.002). PP significantly improved, with TMT A scores decreasing indicating faster speed of completion (*p* = 0.030). VA and LA did not change significantly: ROCF-copy (*p* = 0.483), Animals (*p* = 0.916), and Vegetables (*p* = 0.498). Depressive symptoms, assessed via GDS-15, significantly decreased suggesting elevation of depressive symptoms (*p* = 0.020).

### Comparison between iNPH patients and healthy controls

To assess post-surgery cognitive outcomes, we compared iNPH patients (71.3 ± 5.2 years, 69.7% male) with healthy controls (72.7 ± 4.8 years, 62% male; Table 2). The groups did not significantly differ in demographics. Independent t-tests showed significant cognitive differences favoring the control group (Table 3). MoCA, AVLT I-V, AVLT VI, AVLT VII, ROCF 3 M, ROCF 30 M, TMT B, NKP, Animals, TMT A (all *p* < 0.001), ROCF Copy (*p* = 0.006), and GDS-15 (*p* = 0.004) all showed significant differences, with iNPH patients performing worse. Unlike in previous within subject comparison, we did not use BDT test for assessing Executive Functions in patiants versus controls as the BDT test was not included in neuropsychological protocol for controls.

**Table 2:** Descriptives between included iNPH patients and control group.

Group	n	Age (M ± SD)	Male (%)	Education category*	Number of patients
iNPH	30	71.3 ± 5.2	69.7%	1	1
				2	11
				3	14
				4	7
Control	71	72.7 ± 4.8	62.0%	1	3
				2	23
				3	17
				4	28

*Education categories: 1 = Basic education; 2 = Secondary education without completion certificate; 3 = Secondary education with completion certificate/diploma; 4 = Tertiary (university) education.

**Table 3:** Summary of neuropsychological test performance in iNPH and control groups.

Domain	Test	Group	N	Mean	Median	SD	SE	p_value
Global cognition	MoCA	controls	71	25.73	26	2.20	0.262	<0.001
		iNPH	30	21.35	22	4.36	0.796	
Verbal memory (vM)	AVLT I-V	controls	71	42.94	44	9.45	1.122	<0.001
		iNPH	29	29.62	30	8.70	1.616	
	AVLT VI	controls	71	8.73	9	2.98	0.353	<0.001
		iNPH	29	3.66	4	2.18	0.404	
	AVLT VII	controls	71	7.92	8	3.52	0.418	<0.001
		iNPH	29	3.62	4	2.82	0.524	
Non-verbal memory (nvM)	ROCF 3 min	controls	71	16.64	16,5	5.86	0.695	<0.001
		iNPH	29	11.26	11,5	5.72	1.062	
	ROCF 30 min	controls	71	15.98	15,5	6.26	0.742	<0.001
		iNPH	27	11.19	11,5	5.49	1.057	
Executive functions (EFs)	TMT B	controls	71	124.46	113	56.27	6.678	<0.001
		iNPH	24	196.13	149,5	109.85	22.422	
	NKP	controls	71	41.56	39	10.59	1.256	<0.001
		iNPH	29	28.72	32	10.48	1.947	
Language (LA)	Animals	controls	71	22.32	23	5.74	0.682	<0.001
		iNPH	29	14.9	15	4.50	0.835	
Psychomotor pace (PP)	TMT A	controls	71	50.46	44	22.15	2.629	<0.001
		iNPH	29	75.1	72	29.96	5.564	
Visuospatial abilities (VA)	ROCF copy	controls	71	30.76	31	3.45	0.409	0.006
		iNPH	29	28.05	30	6.18	1.147	
Depressive symptoms	GDS	controls	71	2.11	2	2.32	0.275	0.004
		iNPH	29	3.62	3	2.38	0.442	

For each test, the table reports sample size (N), mean, median, standard deviation (SD), and standard error of the mean (SE) separately for controls and iNPH. Measures where higher scores indicate better performance include MoCA, AVLT (I–V, VI, VII), ROCF (copy, 3-min, 30-min recall), NKP, and category fluency (Animals, Vegetables). Measures where lower values indicate better performance include TMT A/B (completion time) and GDS-15 (depressive symptoms). SE values are derived from the reported SD and N.

## Discussion

We characterized the neuropsychological profile of patients with iNPH and its evolution 3 months after shunt surgery using a multidimensional battery administered pre- and postoperatively, with performance benchmarked against matched healthy controls. The clearest and most consistent signal was a postoperative improvement in vM. Although vM performance remained below that of healthy individuals, the relative gain was larger than in any other cognitive domain. These findings reinforce the notion that vM—particularly facets supported by fronto-subcortical circuitry—may be especially sensitive to CSF diversion in iNPH (Xiao et al., 2022). Our recent work has further suggested that memory impairment in iNPH is uneven across subdomains, a pattern replicated here (Rýdlo et al., 2024). In contrast, nvM did not improve and remained significantly impaired at 3 months. First, nvM may rely on neural systems that are less amenable to short-term physiological normalization after CSF diversion, whether because of greater structural compromise or because improvement unfolds on a longer timeline. Second, nvM tests often have higher demands on visuoperceptual analysis and construction; persistent deficits could therefore reflect a bottleneck outside “pure” memory storage and retrieval. Longitudinal reports are mixed: some show cognitive gains at 12 months, whereas others report transient or absent effects (Büyükgök et al., 2021; Lilja-Lund et al., 2023). Our results fit a model in which nvM either requires a longer recovery window or is comparatively refractory to shunting.

This vM–nvM dissociation may have diagnostic value. In AD, both verbal and non-verbal episodic memory are typically compromised, largely owing to hippocampal atrophy and medial temporal system dysfunction (Bonner-Jackson et al., 2015; Picascia, 2015). In iNPH, by contrast, relative sparing of nvM alongside improved vM could serve as a supportive feature pointing away from primary amnestic pathology. We emphasize, however, that this inference is secondary: we did not include an AD comparison group and thus cannot claim direct discriminative accuracy. Nonetheless, the pattern aligns with literature describing iNPH as a disorder with prominent fronto-subcortical and attention/executive contributions to mnemonic difficulties, as opposed to the medial temporal–dominant deficits seen in AD. Executive functions yielded a mixed picture. Contrary to several prior studies (Duinkerke et al., 2004; Xiao et al., 2022), set-shifting as indexed by TMT-B did not improve at the group level. TMT-B performance is sensitive to baseline variability and to non-executive influences—motor speed, visual search, and motivation—which may obscure moderate improvements. The absence of change may also reflect task insensitivity to the specific executive processes most responsive to CSF diversion. By contrast, we observed a clear improvement on BDT, with the largest effect size across the battery (d = −0.660). Block Design requires visuo-constructive integration andplanning under time pressure—processes strongly linked to dorsolateral prefrontal and parietal systems. While arguably a more demanding overall process, BDT does not require perspective changing and hence does not burden working memory as much as TMT-B does. The profile of gains therefore suggests that structured, planful problem-solving along with visual planning rather than visual attention and action shifting may be more amenable to early postoperative recovery than rapid cognitive flexibility per se (Ogino et al., 2006). Psychomotor processing speed, measured by TMT-A, improved significantly, converging with the view that psychomotor slowing is a core cognitive feature of iNPH and a sensitive marker of treatment response (Xiao et al., 2022). Not all prior studies have found this effect (e.g., Savolainen et al., 2002), likely reflecting heterogeneity in follow-up intervals, inclusion criteria, and analytic methods. Our data indicate that speeded visual–motor integration is responsive by 3 months, consistent with early clinical gains in gait and global functional status.

Language and verbal attention showed no significant postoperative change. Notably, language was among the least impaired domains at baseline and remained relatively preserved compared with healthy controls. This pattern mirrors the broader iNPH cognitive phenotype, in which deficits cluster in attention/executive and memory-related processes, with variable involvement of visuospatial skills and relatively modest language disruption (Saito et al., 2011; Rýdlo et al., 2024). Diffusion tensor imaging studies provide a plausible anatomical rationale: shunt-responsive changes are most evident in periventricular and frontal–subcortical pathways (e.g., corona radiata, corticospinal tracts, and adjacent association fibers) that subserve gait and executive control, while perisylvian language networks are less directly distorted by ventricular enlargement. The lack of language gains may thus reflect both a “floor for improvement,” given baseline preservation, and limited sensitivity of brief language measures to subtle change. Together, these patterns support overall positive neuropsychological outcomes after shunt surgery (Xiao et al., 2022) but also underscore substantial inter-individual variability. Solana et al. (2012) showed that group-level improvement can coexist with fewer than half of individuals meeting criteria for meaningful cognitive gains—an observation with direct clinical consequences. However, positive neurological outcomes have been reported for iNPH patients with presence of preoperative aqueductal CSF flow acceleration on dynamic brain MRI regardless of age, CSF sampling or comorbidities (Bianconi et al., 2024). Nevertheless, identifying predictors and moderators of cognitive response—baseline domain strengths and weaknesses, gait response, imaging markers of white-matter integrity, and comorbid cerebrovascular disease—should be a priority for prospective studies.

Beyond cognition, we observed a small but reliable reduction in depressive symptoms after surgery. Scores remained elevated relative to healthy controls, but improvement from baseline was statistically and clinically meaningful. We caution against attributing a direct “antidepressant” effect to CSF diversion. A more plausible mechanism is indirect mediation: enhanced gait, functional capacity, and health-related quality of life can lessen dysphoria and anergia, which in turn may facilitate better cognitive performance through improved engagement and reduced psychomotor burden. The mean change on GDS-15 was ≈1 point, suggesting modest but meaningful improvement, while setting appropriate expectations (Israelsson et al., 2016). A notable methodological lesson from our data is the limited sensitivity of global screening tools—specifically MoCA—to detect postoperative cognitive change in iNPH. Despite improvements captured by the comprehensive battery, MoCA scores did not shift meaningfully in our cohort. This observation does not contradict the usefulness of MMSE/MoCA in the diagnostic phase: both instruments are recommended by current guidelines and several studies have proposed cut-offs on these scales to define cognitive responders after tap test or extended lumbar drainage (Nakajima et al., 2021; Gallina et al., 2018; Passaretti et al., 2023; Caneva et al., 2025). Rather, our findings suggest that such global measures may fail to register domain-limited but clinically relevant gains, particularly in verbal memory and psychomotor speed. At present, there are no widely accepted responder cut-offs based on standard neuropsychological tests such as AVLT or ROCF, and defining such thresholds was beyond the scope of this study. For both clinical follow-up and trials, we recommend targeted batteries emphasizing attention/executive control, speed, and memory subdomains, complemented by performance-based functional measures.

### Study strengths and limitations

Several limitations merit consideration. First, preoperative CSF tests have imperfect diagnostic accuracy, raising the possibility that some true responders were excluded or that selection bias influences observed effects. Second, our three-month follow-up, while clinically relevant, cannot address the durability, peak timing, or late emergence of cognitive changes. Longer-term trajectories—especially for nvM and higher-order executive functions—require 6- to 12-month reassessment. Third, although our neuropsychological battery balanced comprehensiveness with patient tolerability, several domains were represented by single measures, limiting construct coverage and reliability. The use of raw scores preserves ecological interpretability and avoids distortions from small normative samples but reduces cross-study comparability. Fourth, the sample size was adequate for detecting medium effects at the group level but insufficient for nuanced subgroup analyses (e.g., by cognitive reserve, gait response, comorbidity burden, or symptom duration). Fifth, although practice effects are reported to be minimal in iNPH and unlikely to account for our findings (Peterson et al., 2016), their complete absence cannot be guaranteed. Finally, symptom onset was estimated retrospectively via caregiver reports, introducing recall bias (Krahulik et al., 2020). Counterbalancing these limitations, the inclusion of matched healthy controls strengthens our ability to contextualize postoperative performance and to judge whether changes approach normalization versus reflecting relative improvement from a low baseline. The two-time-point, within-subject design increases sensitivity to change, and the domain-specific emphasis provides a more mechanistic view than global screens alone.

Another limitation is the restricted severity range of our neuropsychological sample. Patients with MoCA scores < 18 or GDS-15 scores ≥ 10 were excluded to avoid floor effects and major mood-related confounds and to ensure that the detailed neuropsychological battery remained interpretable. As a result, our findings primarily generalize to iNPH patients with mild-to-moderate cognitive impairment and relatively low depressive symptom burden. Cognitive trajectories in more severely impaired patients may differ, and treatment effects in this group could be either attenuated (because of limited cognitive reserve) or appear larger on coarse global scales; our study was not designed to address this part of the spectrum.

### Clinical and research implications

Our results support three practical takeaways. First, vM and psychomotor speed are reliable early indicators of cognitive response to shunting. Integrating these markers into routine postoperative follow-up may provide sensitive, time-efficient tracking of recovery. Second, the vM–nvM dissociation has potential diagnostic utility: relative preservation of nvM, alongside improved vM and speed, may help distinguish iNPH from AD in ambiguous cases, particularly when paired with structural and diffusion imaging. Third, reliance on global screens alone risks missing meaningful postoperative change; targeted batteries emphasizing executive–attentional control, speed, and memory subdomains should be preferred for clinical decision-making and for endpoints in trials. Future work should pursue multivariate prognostic models combining neuropsychological features with imaging—DTI measures of periventricular and frontoparietal integrity, callosal angle—CSF biomarkers, gait response, and indices of cognitive reserve. Longitudinal designs extending to 12 months and beyond will be essential to determine whether domains such as nvM and set-shifting show delayed recovery or remain static. Notably, verbal fluency showed a favorable yet non-significant trend (*p* = 0.070), consistent with early but incomplete executive recovery. DTI work demonstrates that shunt-responsive change concentrates in periventricular and frontoparietal pathways, with partial reversibility of tract abnormalities reported in responders (Bubeníková et al., 2025; Kanno et al., 2017; Tang et al., 2021). Clinically, expectation management remains essential: shunting often benefits cognition but trajectories are heterogeneous, and some domains recover slowly or not at all (Solana et al., 2012). Regarding mood, the mean change on GDS-15 was ≈1 point, suggesting modest but meaningful improvement without implying a primary antidepressant effect; indirect mediation via gait and function is plausible (Israelsson et al., 2016). At the measurement level, MoCA’s insensitivity to postoperative change underscores the need for domain-focused follow-up, particularly in attention/executive control, processing speed, and memory subdomains. Furthermore, previous studies have applied heterogeneous diagnostic and follow-up paradigms, sometimes yielding conflicting results regarding cognitive outcomes after CSF diversion (Marmarou et al., 2005), which further complicates the derivation of universally applicable responder criteria.

Therefore, domain-specific assessments, not global screens, should anchor postoperative evaluation and future therapeutic trials.

## Conclusion

The shunt surgery seems to positively influence the neuropsychological performance of patients with iNPH. The most compelling and consistent finding was the significant postoperative improvement in vM, which, although still below normative levels, demonstrated the greatest relative gain among all evaluated cognitive domains. This result underscores the centrality of vM as a surgery-sensitive domain in iNPH and supports the notion that specific aspects of memory—especially those dependent on fronto-subcortical connectivity—are amenable to intervention via CSF diversion. Along with vM, PP also significantly improved, which strengthens the hypothesis that psychomotor speed serves as a sensitive index of functional change and therapeutic responsiveness. Interestingly, mixed results were found for EFs, that contrary to prior findings did not demonstrate improvement in set-shifting ability but improved part of the EFs reliant on structured planning rather than rapid cognitive flexibility. Visuospatial abilities and language remained unimproved by the shunt surgery. It is also noteworthy that none of the cognitive domains declined during the study period, and the absence of deterioration might be interpreted as a positive outcome in itself, even though this inferation exceeds the scope of this study. Beyond cognitive outcomes, our study identified an alleviation of depressive symptoms following shunt surgery. Although depressive symptoms remained elevated in comparison to healthy adults, the reduction from preoperative levels was statistically and clinically significant.

## Data Availability

The raw data supporting the conclusions of this article will be made available by the authors, without undue reservation.

## Footnotes

1
https://www.jamovi.org/
